# Crystal structure of bis­[*cis*-di­aqua­bis­(phen­an­thro­line)cobalt(II)] bis­(citrato)germanate(IV) dinitrate

**DOI:** 10.1107/S205698902100846X

**Published:** 2021-08-20

**Authors:** Olha Buchko, Viktoriya Dyakonenko, Elena Martsinko, Elena Chebanenko

**Affiliations:** a I.I. Mechnikov Odessa National University, 2, Dvoryanskaya str., Odessa, 65082, Ukraine; b SSI "Institute for Single Crystals", National Academy of Sciences of Ukraine, Naukyi Ave. 60, Kharkiv 61001, Ukraine

**Keywords:** crystal structure, bis­(citrato)germanate, 3*d* metal salt, cobalt

## Abstract

The asymmetric unit of the title compound, [Co(C_12_H_8_N_2_)_2_(H_2_O)_2_]_2_[Ge(C_6_H_5_O_7_)_2_](NO_3_)_2_, features two complex [(C_12_H_8_N_2_)_2_(H_2_O)_2_Co]^2+^ cations, two NO_3_^−^ anions as well as one centrosymmetric [(C_6_H_5_O_7_)_2_Ge]^2−^ anion. Two HCit ligands (Cit = citrate, C_6_H_4_O_7_) each coordinate *via* three different oxygen atoms (hy­droxy­late, α-carboxyl­ate, β-carboxyl­ate) to the Ge atom, forming a slightly distorted octa­hedron. The coordination polyhedron of the Co atom is also octa­hedral, formed by coordination of four nitro­gen atoms from two phenanthroline mol­ecules and two water oxygen atoms. In the crystal, the cations and anions are linked by hydrogen bonds and form layers parallel to the *bc* plane. The structure exhibits disorder of the NO_3_^−^ anion [disorder ratio 0.688 (9) to 0.312 (9)]. There are also highly disordered solvent mol­ecules (presumably water and/or ethanol) in the crystal structure; explicit refinement of these mol­ecules was not possible, and the content of the voids was instead taken into account using reverse Fourier transform methods [SQUEEZE procedure in *PLATON*; Spek (2015 ▸). *Acta Cryst*. C**71**, 9–18]. The given chemical formula and other crystal data do not take into account the unknown solvent mol­ecule(s).

## Abstract 2

The mol­ecular and crystal structure of the [Co(H_2_O)_2_(phen)_2_]_2_[Ge(HCit)_2_](NO_3_)_2_ (H_4_Cit is citric acid, phen is 1,10-phenanthroline) compound was studied using X-ray diffraction analysis.

## Chemical context   

Citric acid (H_4_Cit) is an essential component of the Krebs cycle and a universal inter­mediate in plant and animal metab­olism. Its biocompatibility, hydro­philicity and general safety make citric acid a common component in foodstuffs, beverages, pharmaceuticals, cosmetics, etc (Nangare *et al.*, 2021 ▸). Recently, Varbanets and co-workers have reported that germanium coordination compounds with citric acid combined with a second metal and ligand, such as Co and 1,10-phenanthroline (phen), show high anti­hypoxic, cerebroprotective properties and have an activation effect on enzymes (Lukianchuk *et al.*, 2019 ▸; Gudzenko *et al.*, 2019*a* ▸,*b* ▸). Complex compounds have been obtained through reactions in the system GeO_2_–H_4_Cit–Co*X*_2_–phen–C_2_H_5_OH–H_2_O (*X* = Cl, CH_3_COO). The authors reported that the anion of the cobalt salt (chloride and acetate) affects the composition and structure of the complex and results in the formation of cation–anionic compounds such as [Co(phen)_3_][Ge(HCit)_2_]·2H_2_O (Seifullina *et al.*, 2017*a* ▸) or [Co(H_2_O)_2_(phen)_2_]_2_[Ge(Cit)_2_]·4H_2_O (Martsinko *et al.*, 2018*a* ▸,*b* ▸). A bis­(citrato)germanate anion with HCit_3_ ligands tridentately coordinated to germ­anium are implemented in the structure [Co(phen)_3_][Ge(HCit)_2_]·2H_2_O. In the [Co(phen)_3_]^2+^ cation, the cobalt atom binds to three phenanthroline mol­ecules. In [Co(H_2_O)_2_(phen)_2_]_2_[Ge(Cit)_2_]·4H_2_O, on the other hand, cobalt(II) combines with only two mol­ecules of 1,10-phenanthroline and the oxygen atoms of two coordinated water mol­ecules to complete the octa­hedral metal coordination. In this compound, the third carb­oxy­lic group of the citric acid is deprotonated, which leads to a change of the charge of the anion and of the molar Co:Ge ratio, while the coordination polyhedron of the germanium atom remains the same: distorted octa­hedral, formed by six oxygen atoms of three types of oxygen atoms from two tridentate chelating citrate ligands.
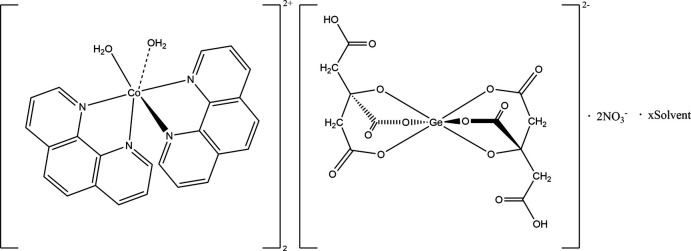


In the present work, we report the synthesis and structural analysis of a new complex, [Co(H_2_O)_2_(phen)_2_]_2_[Ge(HCit)_2_(NO_3_)_2_], which was synthesized by changing the anion of the initial cobalt(II) salt to nitrate. This study is important for establishing the effect that the anion of the 3d metal salt has on the composition and structure of heterometal bis­(citrato)germanates with 1,10-phenanthroline, as well as for the creation of new bioactive compounds.

## Structural commentary   

**Figure 1:**
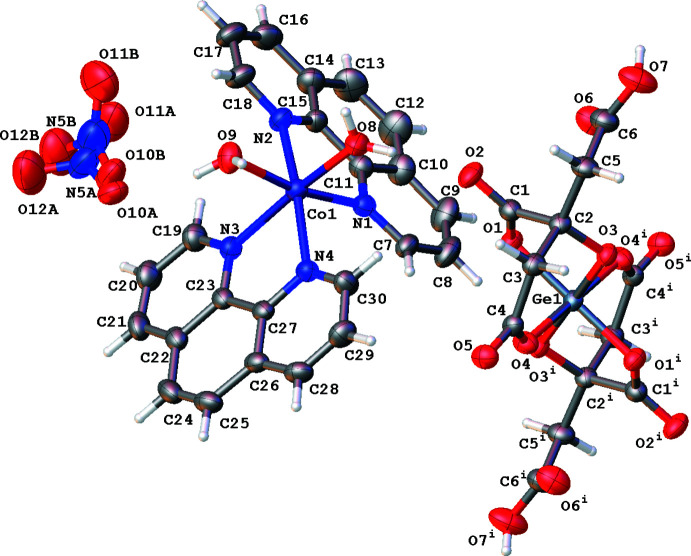
The mol­ecular structure of [Co(H_2_O)_2_(phen)_2_]_2_[Ge(HCit)_2_(NO_3_)_2_] [symmetry code: (i)1 − *x*, 2 − *y*, 1 − *z*].

The title compound is a salt (Fig. 1 ▸), with a complex Co-based cation and two types of anions – the complex anion Ge(HCit)_2_ and nitrate. The Ge atom occupies a special position on an inversion centre [the coordinates are (0.5, 1.0, 0.5)] so only half of the complex anion is located in the asymmetric unit. The charge of the two [Co(H_2_O)_2_(phen)_2_]^2+^ cations are compensated by one Ge complex dianion and two nitrate anions.

The coordination polyhedron of the Ge atom is a distorted octa­hedron formed by oxygen atoms of three types: hydroxyl (O3), α-carboxyl­ate (O1) and β-carboxyl­ate (O4) of two HCit^3−^ ligands. The Ge—O bond lengths are consequently not equivalent. The Ge1—O3 hydroxyl bond [1.813 (2) Å] is shorter than the bonds with the carboxyl­ate oxygen atoms. In addition, the Ge—O1 bond with the α-carboxyl­ate oxygen atom is shorter than the Ge–O4 bond with the β-carboxyl­ate oxygen atom [1.914 (3) Å and 1.959 (3) Å, respectively]. The values of O—Ge—O bond angles lie in the 87.6 (1)–92.4 (1)° range (Table 1 ▸). The structure of the complex germanate anion is in a good agreement with those of similar complexes containing citratogermanates previously described (Martsinko *et al.*, 2013 ▸, 2018*a* ▸,*b* ▸; Seifullina *et al.*, 2017*a* ▸,*b* ▸, 2019 ▸).

The coordination of the organic ligands to the Ge atom forms five- and six-membered metallocycles. The Ge1–O3–C2–C3–C4–O4 six-membered ring adopts a half-chair conformation [the C2 and O3 atoms deviate by 0.277 (4) and −0.657 (3) Å, respectively, from the mean plane though atoms Ge1, C3, C4 and O4, which is planar within 0.01 Å]. The Ge1—O1—C1—C2—O3 five-membered ring adopts an envelope conformation. Atom O3 deviates by 0.527 (3) Å from the mean plane of the remaining ring atoms (planar within 0.03 Å).

The coordination polyhedron of the Co atom is a distorted octa­hedron, which is formed by nitro­gen atoms of two phenanthroline mol­ecules and oxygen atoms of two water mol­ecules. The Co—N and Co—O bond lengths lie in the ranges 2.120 (3)–2.160 (3) and 2.083 (3)–2.098 (3) Å, respectively, while the N—Co—N, O—Co—N and O—Co—O angles are in the range 77.6 (1)–98.6 (1)° (Table 1 ▸).

## Supra­molecular features   

**Figure 2:**
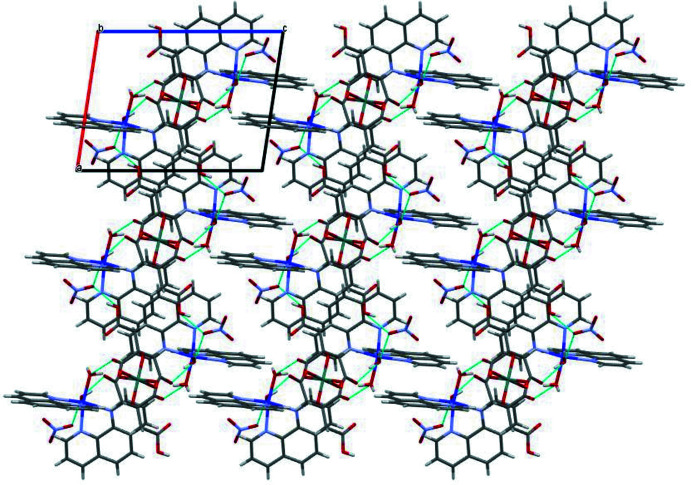
The crystal packing of [Co(H_2_O)_2_(phen)_2_]_2_[Ge(HCit)_2_(NO_3_)_2_] viewed along the *b* axis.

In the crystal, the water mol­ecules of the [Co(H_2_O)_2_(phen)_2_]^2+^ cation are linked to the [Ge(HCit)_2_]^2−^ and NO_3_^−^ anions by inter­molecular O—H⋯O hydrogen bonds (Table 2 ▸); these supra­molecular clusters form layers parallel to the *bc* plane (Fig. 2 ▸). Voids with a volume of 149 Å^3^ containing 49 electrons were found between adjacent layers. The content appears to be a combination of water and ethanol solvent mol­ecules with more than twofold disorder. Refinement of these mol­ecules was not possible, and the content of the voids was instead taken into account using reverse Fourier transform methods (SQUEEZE procedure; Spek, 2015 ▸).

## Database survey   

A search of the Cambridge Structural Database (CSD, Version 5.42, update November 2020; Groom *et al.*, 2016 ▸) for the Ge(HCit)_2_^2−^ anion yielded 15 structures containing this anion (Seiler *et al.*, 2005 ▸; Seifullina *et al.*, 2006 ▸, 2007 ▸, 2015 ▸, 2016 ▸, 2017*a* ▸,*b* ▸; Martsinko *et al.*, 2011 ▸, 2013 ▸, 2018*a* ▸,*b* ▸). In these structures, the Ge—O bond lengths for the hydroxyl, α-carboxyl­ate and β-carboxyl­ate oxygen atoms are in the ranges 1.793–1.840, 1.881–1.914 and 1.904–1.955 Å, respectively.

A search for the [Co(H_2_O)_2_(phen)_2_]^2+^ cation yielded six structures (Batsanov *et al.*, 2011 ▸; Yang *et al.*, 2003 ▸; Bulut *et al.*, 2003 ▸; Abdelhak *et al.*, 2006 ▸; Das *et al.*, 2013 ▸; Fu *et al.*, 2003 ▸). The Co—O bond lengths in the coordination polyhedron vary between 2.073 and 2.140 Å while the Co—N bond lengths range from within 2.118 to 2.164 Å.

No structures containing any combination of [Co(H_2_O)_2_(phen)_2_]^2+^ cations and [Ge(HCit)_2_]^2−^ anions were found in the CSD.

## Synthesis and crystallization   

**Figure 3:**
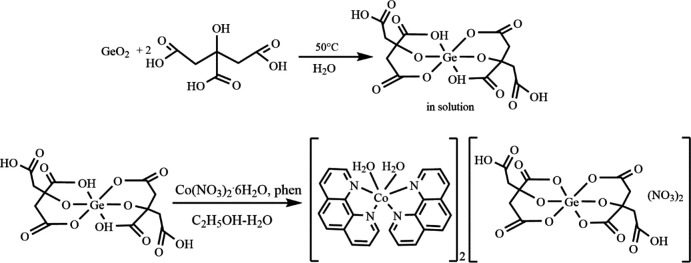
Two-step synthesis of [Co(H_2_O)_2_(phen)_2_]_2_[Ge(HCit)_2_(NO_3_)_2_].

A suspension of germanium(IV) oxide (0.0523 g, 0.5 mmol, GeO_2_, 99.99%, Aldrich) and citric acid (0.21 g, 1 mmol, H_4_Cit·H_2_O, ≥99%, Aldrich) in 100 mL of hot distilled water was stirred to dissolve the reagents completely and slowly evaporated at 323 K to a volume of 20 mL. After cooling the mixture to room temperature, 20 mL of a 95% ethanol solution containing 1,10-phenanthroline (0.18 g, 1 mmol, phen, 99%, Aldrich) and cobalt(II) nitrate hexa­hydrate [0.146 g, 0.5 mmol, Co(NO_3_)_2_·6H_2_O, ≥99%, Aldrich] were added (Fig. 3 ▸). Pink crystals suitable for X-ray analysis were obtained in two days, yield: 63%.

During the study of the thermal stability of the synthesized complex (Q-1500D PerkinElmer), it was established that its decomposition starts with an endothermic peak in the range of 393–423 K (peak 413 K). The corresponding weight loss of 1.5% indicates that the complex includes mol­ecules of solvation. Therefore, crystals were dried at 423 K for 30 min to remove solvate mol­ecules prior to the yield calculation and for elemental analysis.

Analysis calculated for C_60_H_50_Co_2_GeN_10_O_24_ (1485.57) in %: C 48.47, H 3.37, Co 7.94, Ge 4.49, N 9.42; found C 48.25, H 3.26, Co 7.88, Ge 4.35, N 9.40 (ICP optical emission spectrom­eter Optima 2000 DV PerkinElmer and Elemental Analyzer CE-440).

IR (ν_max_, cm^−1^, spectrometer Frontier PerkinElmer, KBr): 3228 ν(OH), 3062, 2917 ν(C—H), 1743 ν(C=O), 1668 ν_as_(COO^−^), 1613 δ(H_2_O), 1587, 1519, 1428 ν(C—C_Ar_), 1410 ν_s_(COO^−^), 1367 ν(C—N), 1089 ν(C—O), 1198, 1148, 915, 856 δ(C—H), 641 ν(Ge—O), 554 ν(Co—O), 425 ν(Co—N).

The IR spectrum of the complex contains absorption bands for ν(C=O), ν_as_(COO^−^) and ν_s_(COO^−^), which indicate the presence of non-equivalent coordinated and free carboxyl groups in the complex. A ν(C—O) absorption band at 1089 cm^−1^ evidences that the alcoholic OH groups of the citrate ligands are deprotonated and involved in coordination. The presence of Ge—O stretching vibrations suggests that the carboxyl­ate and hydroxyl groups are bonded to germanium. Absorption bands assigned to the ν(C—N) heterocycle, the ν(C—C) phenanthroline ring vibrations and deformation vibrations δ(C—H) of the aromatic rings are also found in the IR spectrum. The compound contains coordinated water mol­ecules, as indicated by the H_2_O deformation vibrations at 1613 cm^−1^.

## Refinement   

Crystal data, data collection and structure refinement details are summarized in Table 3 ▸. Carbon-bound and carb­oxy­lic acid H atoms were added in calculated positions with C—H bond lengths of 0.93 Å for C—H, 0.97 Å for CH_2_ and 0.82 Å for O—H bonds. Carb­oxy­lic acid H atoms were allowed to rotate but not to tip to best fit the experimental electron density. Water hydrogen atoms of the metal complex were located from difference-Fourier maps of electron density and their positions were refined with restraints of 0.84 (2) Å for O—H bond distances and 1.36 (2) Å for H⋯H distances. The position of one water H atom (H9*A*) was further restrained based on hydrogen bonding considerations. *U*_iso_(H) were set to *xU*_eq_(C,O), where *x* = 1.5 for hydroxyl groups and water mol­ecules and 1.2 for all other H atoms.

The structure exhibits disorder of the NO_3_^−^ anion. All N—O bond distances were restrained to be similar to each other (within a standard deviation of 0.02 Å) and the distance between oxygen atoms O10*B* and O11*B* was restrained to a target value of 2.200 (4) Å. *U* ^ij^ values of nitrate atoms closer to each other than 2 Å were restrained to be similar to each other (within a standard deviation of 0.02 Å^2^). Subject to these conditions, the disorder ratio refined to 0.688 (9):0.312 (9).

There are also highly disordered solvent mol­ecules (presumably water and/or ethanol) in the crystal structure; explicit refinement of these mol­ecules was not possible, and the content of the voids was instead taken into account using reverse Fourier transform methods (SQUEEZE; Spek, 2015 ▸) as implemented in the program *PLATON* (Spek, 2020 ▸). The voids with a volume of 149 Å^3^ contain 49 electrons.

## Supplementary Material

Crystal structure: contains datablock(s) I. DOI: 10.1107/S205698902100846X/zl5018sup1.cif

Structure factors: contains datablock(s) I. DOI: 10.1107/S205698902100846X/zl5018Isup2.hkl

CCDC reference: 2103184

Additional supporting information:  crystallographic information; 3D view; checkCIF report

## Figures and Tables

**Table 1:** Selected geometric parameters (Å, °)

Ge1—O1	1.914 (3)	Co1—N1	2.136 (3)
Ge1—O3	1.813 (2)	Co1—N2	2.123 (3)
Ge1—O4	1.959 (3)	Co1—N3	2.157 (4)
Co1—O8	2.083 (3)	Co1—N4	2.159 (3)
Co1—O9	2.100 (3)		
			
O1—Ge1—O4^i^	90.92 (12)	O8—Co1—N4	92.07 (14)
O1—Ge1—O4	89.08 (12)	O9—Co1—N2	91.13 (13)
O3—Ge1—O1^i^	92.43 (11)	O9—Co1—N3	88.06 (13)
O3—Ge1—O1	87.58 (11)	O9—Co1—N4	92.60 (13)
O3—Ge1—O4^i^	89.40 (11)	N1—Co1—N3	96.18 (12)
O3—Ge1—O4	90.60 (11)	N1—Co1—N4	98.61 (12)
O8—Co1—O9	86.92 (13)	N2—Co1—N1	78.06 (12)
O8—Co1—N1	90.75 (13)	N2—Co1—N3	94.13 (14)
O8—Co1—N2	96.54 (14)	N3—Co1—N4	77.58 (13)

Symmetry code: (i) -x+1, -y+2, -z+1.

**Table 2:** Hydrogen-bond geometry (Å, °)

*D*—H⋯*A*	*D*—H	H⋯*A*	*D*⋯*A*	*D*—H⋯*A*
O7—H7*A*⋯O10^ii^	0.82	1.88	2.600 (12)	146
O8—H8*A*⋯O2	0.84 (2)	1.88 (2)	2.709 (4)	168 (5)
O9—H9*A*⋯O5^iii^	0.82 (2)	1.96 (2)	2.701 (4)	150 (4)
O9—H9*B*⋯O10	0.82 (2)	1.99 (3)	2.789 (15)	166 (6)

Symmetry codes: (ii) x+1, y+1, z; (iii) -x+1, -y+1, -z+1.

**Table 3:** Experimental details

Crystal data	
Chemical formula	[Co(C_12_H_8_N_2_)_2_(H_2_O)_2_]_2_[Ge(C_6_H_5_O_7_)_2_]NO_3_
*M*_r_	1485.55
Crystal system, space group	Triclinic, *P*\overline{1}
Temperature (K)	294
*a*, *b*, *c* (Å)	10.6719 (5), 11.8089 (4), 14.0901 (7)
α, β, γ (°)	105.697 (4), 94.026 (4), 104.815 (4)
*V* (Å^3^)	1633.98 (13)
*Z*	1
Radiation type	Mo *K*α
μ (mm^−1^)	1.05
Crystal size (mm)	0.5 × 0.4 × 0.2

Data collection	
Diffractometer	Rigaku Oxford Diffraction Xcalibur, Sapphire3
Absorption correction	Multi-scan (*CrysAlis PRO*; Rigaku OD, 2018 ▸)
*T*_min_, *T*_max_	0.167, 1.000
No. of measured, independent and observed [*I* > 2σ(*I*)] reflections	14229, 7497, 4949
*R*_int_	0.071
(sin θ/λ)_max_ (Å^−1^)	0.650

Refinement	
*R*[*F*^2^ > 2σ(*F* ^2^)], *wR*(*F* ^2^), *S*	0.068, 0.192, 0.98
No. of reflections	7497
No. of parameters	489
No. of restraints	131
H-atom treatment	H atoms treated by a mixture of independent and constrained refinement
Δρ_max_, Δρ_min_ (e Å^−3^)	1.09, −0.87

Computer programs: *CrysAlis PRO* (Rigaku OD, 2018 ▸), *SHELXT2018/3* (Sheldrick, 2015*a* ▸), *SHELXL2018/3* (Sheldrick, 2015*b* ▸) and *OLEX2* (Dolomanov *et al.*, 2009 ▸).

## supplementary crystallographic information

### Crystal data

**Table:**

[Co(C_12_H_8_N_2_)_2_(H_2_O)_2_]_2_[Ge(C_6_H_5_O_7_)_2_](NO_3_)_2_	*Z* = 1
*M**_r_* = 1485.55	*F*(000) = 758
Triclinic, *P*1	*D*_x_ = 1.510 Mg m^−^^3^
*a* = 10.6719 (5) Å	Mo *K*α radiation, λ = 0.71073 Å
*b* = 11.8089 (4) Å	Cell parameters from 3558 reflections
*c* = 14.0901 (7) Å	θ = 3.6–26.0°
α = 105.697 (4)°	µ = 1.05 mm^−^^1^
β = 94.026 (4)°	*T* = 294 K
γ = 104.815 (4)°	Block, colourless
*V* = 1633.98 (13) Å^3^	0.5 × 0.4 × 0.2 mm

### Data collection

**Table:**

Rigaku Oxford Diffraction Xcalibur, Sapphire3 diffractometer	7497 independent reflections
Radiation source: fine-focus sealed X-ray tube, Enhance (Mo) X-ray Source	4949 reflections with *I* > 2σ(*I*)
Graphite monochromator	*R*_int_ = 0.071
Detector resolution: 16.1827 pixels mm^-1^	θ_max_ = 27.5°, θ_min_ = 2.9°
ω scans	*h* = −13→13
Absorption correction: multi-scan (CrysAlisPro; Rigaku OD, 2018)	*k* = −13→15
*T*_min_ = 0.167, *T*_max_ = 1.000	*l* = −17→18
14229 measured reflections	

### Refinement

**Table:**

Refinement on *F*^2^	131 restraints
Least-squares matrix: full	Hydrogen site location: mixed
*R*[*F*^2^ > 2σ(*F*^2^)] = 0.068	H atoms treated by a mixture of independent and constrained refinement
*wR*(*F*^2^) = 0.192	*w* = 1/[σ^2^(*F*_o_^2^) + (0.092*P*)^2^] where *P* = (*F*_o_^2^ + 2*F*_c_^2^)/3
*S* = 0.98	(Δ/σ)_max_ < 0.001
7497 reflections	Δρ_max_ = 1.09 e Å^−^^3^
489 parameters	Δρ_min_ = −0.87 e Å^−^^3^

### Special details

**Table:**

Geometry. All esds (except the esd in the dihedral angle between two l.s. planes) are estimated using the full covariance matrix. The cell esds are taken into account individually in the estimation of esds in distances, angles and torsion angles; correlations between esds in cell parameters are only used when they are defined by crystal symmetry. An approximate (isotropic) treatment of cell esds is used for estimating esds involving l.s. planes.

### Fractional atomic coordinates and isotropic or equivalent isotropic displacement parameters (Å^2^)

**Table:**

	*x*	*y*	*z*	*U*_iso_*/*U*_eq_	Occ. (<1)
Ge1	0.500000	1.000000	0.500000	0.03675 (17)	
Co1	0.33663 (5)	0.61981 (4)	0.77746 (4)	0.04432 (18)	
O1	0.5174 (3)	0.9298 (2)	0.6060 (2)	0.0458 (6)	
O2	0.6415 (3)	0.8220 (3)	0.6500 (2)	0.0586 (8)	
O3	0.6711 (2)	1.0110 (2)	0.4908 (2)	0.0425 (6)	
O4	0.4470 (3)	0.8345 (2)	0.4052 (2)	0.0491 (7)	
O5	0.4742 (3)	0.6620 (2)	0.3179 (2)	0.0542 (7)	
O6	0.8227 (3)	1.0894 (3)	0.7076 (3)	0.0752 (10)	
O7	1.0212 (3)	1.0937 (4)	0.6678 (3)	0.0905 (12)	
H7A	1.046289	1.134511	0.726375	0.136*	
O8	0.5332 (3)	0.6796 (3)	0.7618 (3)	0.0618 (8)	
H8A	0.574 (5)	0.717 (5)	0.725 (4)	0.093*	
H8B	0.588 (4)	0.655 (5)	0.791 (4)	0.093*	
O9	0.3742 (4)	0.4581 (3)	0.7902 (3)	0.0634 (8)	
H9A	0.415 (4)	0.437 (4)	0.745 (3)	0.095*	
H9B	0.306 (3)	0.407 (4)	0.789 (4)	0.095*	
O10	0.1634 (11)	0.2778 (14)	0.8150 (6)	0.079 (2)	0.688 (9)
O10B	0.186 (3)	0.285 (3)	0.8413 (15)	0.105 (6)	0.312 (9)
O11	0.1943 (13)	0.3850 (8)	0.9768 (7)	0.145 (4)	0.688 (9)
O11B	0.287 (2)	0.3400 (17)	0.9953 (13)	0.154 (6)	0.312 (9)
O12	0.0517 (10)	0.2005 (10)	0.9183 (8)	0.157 (4)	0.688 (9)
O12B	0.072 (2)	0.304 (2)	0.9734 (17)	0.151 (5)	0.312 (9)
N1	0.3167 (3)	0.7994 (3)	0.7946 (2)	0.0457 (7)	
N2	0.3626 (4)	0.6943 (3)	0.9350 (2)	0.0506 (8)	
N3	0.1305 (3)	0.5296 (3)	0.7653 (3)	0.0479 (8)	
N4	0.2778 (3)	0.5440 (3)	0.6181 (2)	0.0461 (8)	
N5	0.1351 (12)	0.2883 (9)	0.9041 (7)	0.102 (3)	0.688 (9)
N5B	0.180 (2)	0.319 (3)	0.9359 (18)	0.124 (4)	0.312 (9)
C1	0.6179 (4)	0.8863 (3)	0.6003 (3)	0.0447 (9)	
C2	0.7002 (4)	0.9130 (3)	0.5205 (3)	0.0397 (8)	
C3	0.6587 (4)	0.7976 (4)	0.4312 (3)	0.0454 (9)	
H3A	0.715808	0.809487	0.381935	0.054*	
H3B	0.672611	0.729839	0.452798	0.054*	
C4	0.5174 (4)	0.7610 (3)	0.3810 (3)	0.0394 (8)	
C5	0.8460 (4)	0.9469 (4)	0.5569 (3)	0.0525 (10)	
H5A	0.867066	0.874970	0.566046	0.063*	
H5B	0.893737	0.970051	0.505709	0.063*	
C6	0.8925 (5)	1.0500 (4)	0.6529 (4)	0.0632 (12)	
C7	0.3017 (4)	0.8521 (4)	0.7247 (3)	0.0541 (10)	
H7	0.299949	0.808312	0.658670	0.065*	
C8	0.2886 (5)	0.9685 (5)	0.7449 (5)	0.0770 (16)	
H8	0.277307	1.002198	0.693535	0.092*	
C9	0.2924 (6)	1.0338 (5)	0.8411 (5)	0.0803 (17)	
H9	0.282433	1.112360	0.855452	0.096*	
C10	0.3110 (6)	0.9843 (4)	0.9186 (4)	0.0690 (14)	
C11	0.3231 (4)	0.8637 (4)	0.8909 (3)	0.0497 (10)	
C12	0.3176 (7)	1.0460 (5)	1.0206 (5)	0.092 (2)	
H12	0.309794	1.125379	1.039407	0.110*	
C13	0.3359 (7)	0.9894 (5)	1.0937 (4)	0.0885 (18)	
H13	0.337511	1.030144	1.160419	0.106*	
C14	0.3519 (5)	0.8692 (4)	1.0659 (4)	0.0666 (13)	
C15	0.3463 (4)	0.8074 (4)	0.9668 (3)	0.0486 (9)	
C16	0.3762 (6)	0.8107 (5)	1.1371 (4)	0.0761 (15)	
H16	0.379325	0.848450	1.204675	0.091*	
C17	0.3953 (6)	0.6961 (6)	1.1045 (4)	0.0787 (15)	
H17	0.412778	0.655870	1.149965	0.094*	
C18	0.3882 (5)	0.6420 (5)	1.0041 (4)	0.0684 (13)	
H18	0.401944	0.564997	0.983189	0.082*	
C19	0.0581 (5)	0.5253 (4)	0.8367 (4)	0.0620 (12)	
H19	0.093641	0.575016	0.901218	0.074*	
C20	−0.0684 (5)	0.4500 (5)	0.8195 (5)	0.0702 (13)	
H20	−0.116577	0.450418	0.871997	0.084*	
C21	−0.1230 (5)	0.3757 (5)	0.7271 (5)	0.0674 (13)	
H21	−0.208339	0.324658	0.715527	0.081*	
C22	−0.0488 (4)	0.3766 (4)	0.6486 (4)	0.0531 (10)	
C23	0.0781 (4)	0.4572 (3)	0.6715 (3)	0.0440 (9)	
C24	−0.0971 (5)	0.3013 (4)	0.5461 (4)	0.0644 (13)	
H24	−0.180876	0.246614	0.530428	0.077*	
C25	−0.0238 (5)	0.3089 (4)	0.4742 (4)	0.0634 (12)	
H25	−0.057055	0.259283	0.409219	0.076*	
C26	0.1055 (4)	0.3925 (4)	0.4953 (3)	0.0508 (10)	
C27	0.1561 (4)	0.4645 (3)	0.5929 (3)	0.0436 (9)	
C28	0.1845 (5)	0.4073 (4)	0.4225 (4)	0.0593 (12)	
H28	0.154207	0.361152	0.356240	0.071*	
C29	0.3026 (5)	0.4868 (4)	0.4473 (4)	0.0642 (12)	
H29	0.355017	0.497089	0.398522	0.077*	
C30	0.3479 (5)	0.5546 (4)	0.5458 (3)	0.0555 (10)	
H30	0.431305	0.610072	0.561765	0.067*	

### Atomic displacement parameters (Å^2^)

**Table:**

	*U*^11^	*U*^22^	*U*^33^	*U*^12^	*U*^13^	*U*^23^
Ge1	0.0349 (3)	0.0352 (3)	0.0442 (3)	0.0129 (2)	0.0064 (2)	0.0156 (2)
Co1	0.0491 (3)	0.0407 (3)	0.0417 (3)	0.0123 (2)	0.0033 (2)	0.0112 (2)
O1	0.0451 (15)	0.0529 (15)	0.0486 (16)	0.0186 (13)	0.0101 (13)	0.0250 (12)
O2	0.0620 (19)	0.0699 (19)	0.065 (2)	0.0305 (16)	0.0121 (16)	0.0419 (16)
O3	0.0422 (15)	0.0426 (14)	0.0503 (16)	0.0154 (12)	0.0083 (12)	0.0230 (12)
O4	0.0410 (14)	0.0452 (15)	0.0594 (18)	0.0166 (12)	−0.0003 (13)	0.0106 (12)
O5	0.0590 (18)	0.0459 (15)	0.0540 (18)	0.0197 (14)	0.0005 (14)	0.0068 (13)
O6	0.061 (2)	0.080 (2)	0.073 (2)	0.0225 (18)	0.0113 (19)	0.0010 (18)
O7	0.052 (2)	0.111 (3)	0.084 (3)	0.016 (2)	−0.008 (2)	−0.001 (2)
O8	0.0465 (18)	0.072 (2)	0.069 (2)	0.0075 (16)	−0.0001 (16)	0.0349 (16)
O9	0.071 (2)	0.0506 (17)	0.079 (2)	0.0247 (16)	0.0242 (19)	0.0254 (16)
O10	0.093 (5)	0.080 (4)	0.061 (4)	0.019 (4)	−0.013 (4)	0.026 (4)
O10B	0.157 (11)	0.077 (7)	0.070 (9)	0.009 (9)	−0.009 (9)	0.034 (8)
O11	0.212 (9)	0.093 (5)	0.091 (5)	0.002 (5)	−0.003 (6)	0.012 (4)
O11B	0.175 (12)	0.088 (9)	0.159 (11)	0.000 (9)	−0.015 (10)	0.015 (8)
O12	0.169 (7)	0.153 (7)	0.123 (7)	−0.016 (6)	0.035 (6)	0.053 (6)
O12B	0.167 (10)	0.141 (10)	0.122 (9)	0.011 (9)	0.058 (8)	0.025 (8)
N1	0.0435 (18)	0.0484 (18)	0.0450 (19)	0.0124 (15)	0.0061 (15)	0.0143 (14)
N2	0.061 (2)	0.0498 (19)	0.044 (2)	0.0171 (17)	0.0063 (17)	0.0185 (15)
N3	0.0477 (19)	0.0493 (18)	0.049 (2)	0.0128 (15)	0.0088 (16)	0.0196 (15)
N4	0.0485 (19)	0.0437 (17)	0.0434 (19)	0.0106 (15)	0.0007 (15)	0.0125 (13)
N5	0.151 (7)	0.087 (5)	0.070 (5)	0.029 (5)	0.001 (5)	0.036 (4)
N5B	0.164 (8)	0.104 (7)	0.092 (8)	0.012 (7)	0.015 (7)	0.036 (7)
C1	0.040 (2)	0.041 (2)	0.054 (2)	0.0126 (17)	0.0013 (18)	0.0151 (17)
C2	0.0343 (18)	0.0420 (19)	0.048 (2)	0.0176 (16)	0.0053 (16)	0.0163 (16)
C3	0.041 (2)	0.050 (2)	0.050 (2)	0.0176 (18)	0.0082 (18)	0.0178 (17)
C4	0.045 (2)	0.0319 (17)	0.041 (2)	0.0123 (16)	0.0027 (16)	0.0090 (14)
C5	0.041 (2)	0.056 (2)	0.059 (3)	0.0181 (19)	−0.0031 (19)	0.0122 (19)
C6	0.051 (3)	0.065 (3)	0.066 (3)	0.014 (2)	−0.005 (2)	0.014 (2)
C7	0.051 (2)	0.063 (3)	0.058 (3)	0.017 (2)	0.006 (2)	0.033 (2)
C8	0.080 (4)	0.089 (4)	0.093 (4)	0.038 (3)	0.023 (3)	0.061 (3)
C9	0.102 (4)	0.067 (3)	0.098 (4)	0.047 (3)	0.031 (4)	0.042 (3)
C10	0.079 (4)	0.056 (3)	0.079 (4)	0.031 (3)	0.019 (3)	0.018 (2)
C11	0.050 (2)	0.045 (2)	0.052 (2)	0.0158 (19)	0.0063 (19)	0.0102 (17)
C12	0.126 (6)	0.064 (3)	0.085 (4)	0.042 (4)	0.024 (4)	0.006 (3)
C13	0.110 (5)	0.080 (4)	0.062 (4)	0.030 (3)	0.020 (3)	−0.004 (3)
C14	0.074 (3)	0.065 (3)	0.055 (3)	0.016 (3)	0.005 (3)	0.013 (2)
C15	0.052 (2)	0.047 (2)	0.041 (2)	0.0083 (18)	0.0039 (18)	0.0119 (16)
C16	0.074 (3)	0.096 (4)	0.050 (3)	0.016 (3)	0.004 (3)	0.016 (3)
C17	0.081 (4)	0.107 (4)	0.058 (3)	0.026 (3)	0.002 (3)	0.043 (3)
C18	0.084 (4)	0.077 (3)	0.050 (3)	0.030 (3)	−0.002 (3)	0.025 (2)
C19	0.057 (3)	0.063 (3)	0.068 (3)	0.015 (2)	0.015 (2)	0.024 (2)
C20	0.059 (3)	0.079 (3)	0.083 (4)	0.019 (3)	0.024 (3)	0.038 (3)
C21	0.044 (3)	0.066 (3)	0.100 (4)	0.008 (2)	0.013 (3)	0.044 (3)
C22	0.044 (2)	0.046 (2)	0.073 (3)	0.0119 (18)	0.000 (2)	0.026 (2)
C23	0.040 (2)	0.0384 (19)	0.057 (2)	0.0131 (16)	0.0001 (18)	0.0189 (16)
C24	0.047 (3)	0.049 (2)	0.089 (4)	0.004 (2)	−0.013 (3)	0.021 (2)
C25	0.065 (3)	0.051 (2)	0.062 (3)	0.013 (2)	−0.017 (2)	0.007 (2)
C26	0.055 (2)	0.043 (2)	0.051 (2)	0.0136 (18)	−0.010 (2)	0.0129 (17)
C27	0.043 (2)	0.0398 (19)	0.050 (2)	0.0133 (16)	−0.0026 (18)	0.0167 (16)
C28	0.065 (3)	0.058 (3)	0.047 (3)	0.020 (2)	−0.009 (2)	0.0059 (19)
C29	0.078 (3)	0.070 (3)	0.050 (3)	0.026 (3)	0.014 (2)	0.021 (2)
C30	0.054 (3)	0.059 (3)	0.048 (3)	0.008 (2)	0.010 (2)	0.0139 (19)

### Geometric parameters (Å, º)

**Table:**

Ge1—O1^i^	1.914 (3)	C5—H5A	0.9700
Ge1—O1	1.914 (3)	C5—H5B	0.9700
Ge1—O3^i^	1.813 (2)	C5—C6	1.505 (6)
Ge1—O3	1.813 (2)	C7—H7	0.9300
Ge1—O4^i^	1.959 (3)	C7—C8	1.372 (6)
Ge1—O4	1.959 (3)	C8—H8	0.9300
Co1—O8	2.083 (3)	C8—C9	1.357 (8)
Co1—O9	2.100 (3)	C9—H9	0.9300
Co1—N1	2.136 (3)	C9—C10	1.396 (7)
Co1—N2	2.123 (3)	C10—C11	1.414 (6)
Co1—N3	2.157 (4)	C10—C12	1.411 (8)
Co1—N4	2.159 (3)	C11—C15	1.442 (6)
O1—C1	1.301 (4)	C12—H12	0.9300
O2—C1	1.221 (4)	C12—C13	1.399 (9)
O3—C2	1.429 (4)	C13—H13	0.9300
O4—C4	1.282 (4)	C13—C14	1.425 (7)
O5—C4	1.214 (4)	C14—C15	1.378 (6)
O6—C6	1.196 (5)	C14—C16	1.408 (7)
O7—H7A	0.8200	C16—H16	0.9300
O7—C6	1.317 (6)	C16—C17	1.378 (7)
O8—H8A	0.839 (19)	C17—H17	0.9300
O8—H8B	0.846 (19)	C17—C18	1.374 (7)
O9—H9A	0.821 (17)	C18—H18	0.9300
O9—H9B	0.815 (19)	C19—H19	0.9300
O10—N5	1.292 (10)	C19—C20	1.376 (7)
O10B—N5B	1.294 (14)	C20—H20	0.9300
O11—N5	1.288 (10)	C20—C21	1.348 (8)
O11B—N5B	1.292 (14)	C21—H21	0.9300
O12—N5	1.253 (10)	C21—C22	1.405 (7)
O12B—N5B	1.295 (14)	C22—C23	1.399 (6)
N1—C7	1.321 (5)	C22—C24	1.452 (7)
N1—C11	1.351 (5)	C23—C27	1.440 (5)
N2—C15	1.349 (5)	C24—H24	0.9300
N2—C18	1.334 (5)	C24—C25	1.331 (7)
N3—C19	1.314 (5)	C25—H25	0.9300
N3—C23	1.353 (5)	C25—C26	1.433 (7)
N4—C27	1.351 (5)	C26—C27	1.391 (6)
N4—C30	1.321 (5)	C26—C28	1.392 (6)
C1—C2	1.524 (5)	C28—H28	0.9300
C2—C3	1.524 (5)	C28—C29	1.322 (7)
C2—C5	1.518 (5)	C29—H29	0.9300
C3—H3A	0.9700	C29—C30	1.381 (6)
C3—H3B	0.9700	C30—H30	0.9300
C3—C4	1.521 (5)		
			
O1^i^—Ge1—O1	180.0	C6—C5—C2	114.2 (3)
O1^i^—Ge1—O4^i^	89.08 (12)	C6—C5—H5A	108.7
O1—Ge1—O4^i^	90.92 (12)	C6—C5—H5B	108.7
O1^i^—Ge1—O4	90.92 (12)	O6—C6—O7	124.1 (5)
O1—Ge1—O4	89.08 (12)	O6—C6—C5	125.0 (4)
O3—Ge1—O1^i^	92.43 (11)	O7—C6—C5	110.9 (4)
O3^i^—Ge1—O1^i^	87.57 (11)	N1—C7—H7	118.5
O3—Ge1—O1	87.58 (11)	N1—C7—C8	123.0 (5)
O3^i^—Ge1—O1	92.43 (11)	C8—C7—H7	118.5
O3^i^—Ge1—O3	180.0	C7—C8—H8	120.5
O3^i^—Ge1—O4^i^	90.60 (11)	C9—C8—C7	119.0 (5)
O3—Ge1—O4^i^	89.40 (11)	C9—C8—H8	120.5
O3^i^—Ge1—O4	89.40 (11)	C8—C9—H9	119.6
O3—Ge1—O4	90.60 (11)	C8—C9—C10	120.8 (4)
O4—Ge1—O4^i^	180.0	C10—C9—H9	119.6
O8—Co1—O9	86.92 (13)	C9—C10—C11	116.4 (5)
O8—Co1—N1	90.75 (13)	C9—C10—C12	124.6 (5)
O8—Co1—N2	96.54 (14)	C12—C10—C11	119.0 (5)
O8—Co1—N3	168.28 (14)	N1—C11—C10	121.9 (4)
O8—Co1—N4	92.07 (14)	N1—C11—C15	118.3 (3)
O9—Co1—N1	168.62 (14)	C10—C11—C15	119.8 (4)
O9—Co1—N2	91.13 (13)	C10—C12—H12	119.6
O9—Co1—N3	88.06 (13)	C13—C12—C10	120.9 (5)
O9—Co1—N4	92.60 (13)	C13—C12—H12	119.6
N1—Co1—N3	96.18 (12)	C12—C13—H13	120.0
N1—Co1—N4	98.61 (12)	C12—C13—C14	120.0 (5)
N2—Co1—N1	78.06 (12)	C14—C13—H13	120.0
N2—Co1—N3	94.13 (14)	C15—C14—C13	120.1 (5)
N2—Co1—N4	170.78 (13)	C15—C14—C16	117.9 (4)
N3—Co1—N4	77.58 (13)	C16—C14—C13	122.0 (5)
C1—O1—Ge1	109.6 (2)	N2—C15—C11	116.4 (4)
C2—O3—Ge1	107.7 (2)	N2—C15—C14	123.4 (4)
C4—O4—Ge1	127.8 (3)	C14—C15—C11	120.2 (4)
C6—O7—H7A	109.5	C14—C16—H16	120.7
Co1—O8—H8A	134 (3)	C17—C16—C14	118.5 (5)
Co1—O8—H8B	118 (3)	C17—C16—H16	120.7
H8A—O8—H8B	107 (3)	C16—C17—H17	120.3
Co1—O9—H9A	106 (3)	C18—C17—C16	119.3 (5)
Co1—O9—H9B	110 (4)	C18—C17—H17	120.3
H9A—O9—H9B	114 (3)	N2—C18—C17	123.3 (5)
C7—N1—Co1	128.3 (3)	N2—C18—H18	118.3
C7—N1—C11	118.9 (4)	C17—C18—H18	118.3
C11—N1—Co1	112.8 (3)	N3—C19—H19	118.7
C15—N2—Co1	114.3 (3)	N3—C19—C20	122.5 (5)
C18—N2—Co1	128.2 (3)	C20—C19—H19	118.7
C18—N2—C15	117.5 (4)	C19—C20—H20	119.7
C19—N3—Co1	128.8 (3)	C21—C20—C19	120.5 (5)
C19—N3—C23	118.5 (4)	C21—C20—H20	119.7
C23—N3—Co1	112.2 (3)	C20—C21—H21	120.5
C27—N4—Co1	112.8 (3)	C20—C21—C22	118.9 (4)
C30—N4—Co1	129.2 (3)	C22—C21—H21	120.5
C30—N4—C27	117.6 (4)	C21—C22—C24	124.0 (4)
O11—N5—O10	120.7 (11)	C23—C22—C21	117.3 (4)
O12—N5—O10	118.2 (10)	C23—C22—C24	118.7 (4)
O12—N5—O11	121.1 (10)	N3—C23—C22	122.3 (4)
O10B—N5B—O12B	124 (3)	N3—C23—C27	118.4 (3)
O11B—N5B—O10B	116.8 (15)	C22—C23—C27	119.3 (4)
O11B—N5B—O12B	117 (2)	C22—C24—H24	119.3
O1—C1—C2	115.1 (3)	C25—C24—C22	121.3 (4)
O2—C1—O1	123.4 (4)	C25—C24—H24	119.3
O2—C1—C2	121.4 (3)	C24—C25—H25	119.5
O3—C2—C1	109.2 (3)	C24—C25—C26	121.0 (4)
O3—C2—C3	108.5 (3)	C26—C25—H25	119.5
O3—C2—C5	109.4 (3)	C27—C26—C25	119.4 (4)
C3—C2—C1	106.9 (3)	C27—C26—C28	117.2 (4)
C5—C2—C1	112.1 (3)	C28—C26—C25	123.4 (4)
C5—C2—C3	110.6 (3)	N4—C27—C23	117.3 (3)
C2—C3—H3A	108.3	N4—C27—C26	122.4 (4)
C2—C3—H3B	108.3	C26—C27—C23	120.2 (4)
H3A—C3—H3B	107.4	C26—C28—H28	119.9
C4—C3—C2	115.8 (3)	C29—C28—C26	120.1 (4)
C4—C3—H3A	108.3	C29—C28—H28	119.9
C4—C3—H3B	108.3	C28—C29—H29	120.0
O4—C4—C3	119.9 (3)	C28—C29—C30	119.9 (5)
O5—C4—O4	121.5 (4)	C30—C29—H29	120.0
O5—C4—C3	118.6 (3)	N4—C30—C29	122.7 (4)
C2—C5—H5A	108.7	N4—C30—H30	118.7
C2—C5—H5B	108.7	C29—C30—H30	118.7
H5A—C5—H5B	107.6		
			
Ge1—O1—C1—O2	−169.8 (3)	C9—C10—C11—C15	178.3 (5)
Ge1—O1—C1—C2	5.7 (4)	C9—C10—C12—C13	179.6 (6)
Ge1—O3—C2—C1	−32.7 (3)	C10—C11—C15—N2	−177.1 (4)
Ge1—O3—C2—C3	83.5 (3)	C10—C11—C15—C14	2.3 (7)
Ge1—O3—C2—C5	−155.8 (3)	C10—C12—C13—C14	1.9 (11)
Ge1—O4—C4—O5	−177.0 (3)	C11—N1—C7—C8	−1.9 (7)
Ge1—O4—C4—C3	2.6 (5)	C11—C10—C12—C13	0.0 (10)
Co1—N1—C7—C8	179.4 (4)	C12—C10—C11—N1	179.5 (5)
Co1—N1—C11—C10	−179.4 (4)	C12—C10—C11—C15	−2.0 (8)
Co1—N1—C11—C15	2.1 (5)	C12—C13—C14—C15	−1.6 (10)
Co1—N2—C15—C11	−4.1 (5)	C12—C13—C14—C16	177.2 (6)
Co1—N2—C15—C14	176.5 (4)	C13—C14—C15—N2	178.9 (5)
Co1—N2—C18—C17	−175.9 (4)	C13—C14—C15—C11	−0.4 (8)
Co1—N3—C19—C20	−170.6 (3)	C13—C14—C16—C17	−177.7 (6)
Co1—N3—C23—C22	170.9 (3)	C14—C16—C17—C18	−0.9 (9)
Co1—N3—C23—C27	−9.5 (4)	C15—N2—C18—C17	1.7 (8)
Co1—N4—C27—C23	9.6 (4)	C15—C14—C16—C17	1.1 (9)
Co1—N4—C27—C26	−171.0 (3)	C16—C14—C15—N2	0.1 (8)
Co1—N4—C30—C29	170.5 (3)	C16—C14—C15—C11	−179.3 (5)
O1^i^—Ge1—O3—C2	−149.9 (2)	C16—C17—C18—N2	−0.5 (9)
O1—Ge1—O3—C2	30.1 (2)	C18—N2—C15—C11	178.0 (4)
O1—C1—C2—O3	17.6 (4)	C18—N2—C15—C14	−1.5 (7)
O1—C1—C2—C3	−99.6 (4)	C19—N3—C23—C22	−1.4 (5)
O1—C1—C2—C5	139.1 (3)	C19—N3—C23—C27	178.2 (3)
O2—C1—C2—O3	−166.8 (4)	C19—C20—C21—C22	−0.2 (7)
O2—C1—C2—C3	76.0 (5)	C20—C21—C22—C23	−1.0 (6)
O2—C1—C2—C5	−45.3 (5)	C20—C21—C22—C24	−179.7 (4)
O3—C2—C3—C4	−54.0 (4)	C21—C22—C23—N3	1.8 (6)
O3—C2—C5—C6	68.0 (5)	C21—C22—C23—C27	−177.8 (3)
O4—Ge1—O3—C2	−59.0 (2)	C21—C22—C24—C25	177.8 (4)
O4^i^—Ge1—O3—C2	121.0 (2)	C22—C23—C27—N4	179.6 (3)
N1—C7—C8—C9	0.7 (8)	C22—C23—C27—C26	0.2 (5)
N1—C11—C15—N2	1.4 (6)	C22—C24—C25—C26	−0.3 (7)
N1—C11—C15—C14	−179.2 (4)	C23—N3—C19—C20	0.1 (6)
N3—C19—C20—C21	0.7 (8)	C23—C22—C24—C25	−0.9 (6)
N3—C23—C27—N4	0.0 (5)	C24—C22—C23—N3	−179.4 (3)
N3—C23—C27—C26	−179.4 (3)	C24—C22—C23—C27	1.0 (5)
C1—C2—C3—C4	63.7 (4)	C24—C25—C26—C27	1.5 (6)
C1—C2—C5—C6	−53.3 (5)	C24—C25—C26—C28	−177.7 (4)
C2—C3—C4—O4	9.6 (5)	C25—C26—C27—N4	179.2 (3)
C2—C3—C4—O5	−170.7 (3)	C25—C26—C27—C23	−1.4 (5)
C2—C5—C6—O6	13.3 (7)	C25—C26—C28—C29	179.5 (4)
C2—C5—C6—O7	−165.2 (4)	C26—C28—C29—C30	0.5 (7)
C3—C2—C5—C6	−172.5 (4)	C27—N4—C30—C29	−1.1 (6)
C5—C2—C3—C4	−174.0 (3)	C27—C26—C28—C29	0.3 (6)
C7—N1—C11—C10	1.6 (6)	C28—C26—C27—N4	−1.6 (5)
C7—N1—C11—C15	−176.9 (4)	C28—C26—C27—C23	177.8 (3)
C7—C8—C9—C10	0.8 (9)	C28—C29—C30—N4	−0.1 (7)
C8—C9—C10—C11	−1.0 (9)	C30—N4—C27—C23	−177.4 (3)
C8—C9—C10—C12	179.3 (6)	C30—N4—C27—C26	2.0 (5)
C9—C10—C11—N1	−0.2 (7)		

Symmetry code: (i) −*x*+1, −*y*+2, −*z*+1.

### Hydrogen-bond geometry (Å, º)

**Table:**

*D*—H···*A*	*D*—H	H···*A*	*D*···*A*	*D*—H···*A*
O7—H7*A*···O10^ii^	0.82	1.88	2.600 (12)	146
O8—H8*A*···O2	0.84 (2)	1.88 (2)	2.709 (4)	168 (5)
O9—H9*A*···O5^iii^	0.82 (2)	1.96 (2)	2.701 (4)	150 (4)
O9—H9*B*···O10	0.82 (2)	1.99 (3)	2.789 (15)	166 (6)

Symmetry codes: (ii) *x*+1, *y*+1, *z*; (iii) −*x*+1, −*y*+1, −*z*+1.
